# Canine Primary Intracranial Cancer: A Clinicopathologic and Comparative Review of Glioma, Meningioma, and Choroid Plexus Tumors

**DOI:** 10.3389/fonc.2019.01151

**Published:** 2019-11-08

**Authors:** Andrew D. Miller, C. Ryan Miller, John H. Rossmeisl

**Affiliations:** ^1^Section of Anatomic Pathology, Department of Biomedical Sciences, Cornell University College of Veterinary Medicine, Ithaca, NY, United States; ^2^Division of Neuropathology, Department of Pathology, O'Neal Comprehensive Cancer Center and Comprehensive Neuroscience Center, University of Alabama School of Medicine, Birmingham, AL, United States; ^3^Section of Neurology and Neurosurgery, Veterinary and Comparative Neuro-Oncology Laboratory, Department of Clinical Sciences, Virginia-Maryland College of Veterinary Medicine, Blacksburg, VA, United States

**Keywords:** canine, glioma, meningioma, choroid plexus tumor, pathology, immunohistochemistry

## Abstract

In the dog, primary intracranial neoplasia represents ~2–5% of all cancers and is especially common in certain breeds including English and French bulldogs and Boxers. The most common types of primary intracranial cancer in the dog are meningioma, glioma, and choroid plexus tumors, generally occurring in middle aged to older dogs. Much work has recently been done to understand the characteristic imaging and clinicopathologic features of these tumors. The gross and histologic landscape of these tumors in the dog compare favorably to their human counterparts with many similarities noted in histologic patterns, subtype, and grades. Data informing the underlying molecular abnormalities in the canine tumors have only begun to be unraveled, but reveal similar pathways are mutated between canine and human primary intracranial neoplasia. This review will provide an overview of the clinicopathologic features of the three most common forms of primary intracranial cancer in the dog, delve into the comparative aspects between the dog and human neoplasms, and provide an introduction to current standard of care while also highlighting novel, experimental treatments that may help bridge the gap between canine and human cancer therapies.

## Introduction

Dogs are the only mammalian species besides humans in which spontaneous brain tumors arise frequently (1–4). The estimated incidence of canine nervous system tumors is reported as 14.5 cases per 100,000 (5). Other studies indicate that intracranial neoplasms are observed in 2–4.5% of dogs that are subjected to post-mortem examinations (2, 4, 5).

In dogs, ~90% of primary brain tumors (PBT) encountered in clinical practice are represented by meningiomas (~50%), gliomas (~35%), and choroid plexus tumors (CPT; ~7%), although the distribution of specific PBT in individual studies varies considerably (1–5). Other PBT, including ependymoma, germinoma, and embryonal tumors are all extremely rare, poorly defined outside of scattered case reports and series, and will not be considered in this review. Secondary brain tumors (SBT) comprise approximately one-half of all canine intracranial tumors, with hemangiosarcoma (29–35%), lymphoma (12–20%), and metastatic carcinomas (11–20%) accounting for 77–86% of all SBT (4, 6).

Brain tumors in dogs occur at any age and in any breed, and there are no reported sex predispositions. However, most PBT and SBT occur in middle-aged to older dogs, with the majority of cases described being > 5 years of age (3, 4, 7, 8). Median ages at diagnosis for dogs with gliomas, meningiomas, and CPT are 8 years, 10.5 years, and 5.5 years, respectively (3, 4, 7, 8). There is a propensity for intracranial tumors identified in juvenile animals to be neuroepithelial tumors of glial, neuronal, or embryonal origin (4, 9). One study identified a statistically significant linear relationship between age and body weight and the occurrence of PBT, and large breed dogs were at significantly increased risk for developing meningiomas and CPT (4). Golden retrievers, boxers, miniature schnauzers, and rat terriers have been identified as breeds in which intracranial meningiomas are overrepresented (3, 4).

Although CPT were also overrepresented in Golden Retrievers in one study (8), this breed predisposition was not substantiated in a subsequent investigation (4). Gliomas (astrocytomas, oligodendrogliomas, and undefined gliomas) are highly overrepresented in several brachycephalic breeds including boxers, Boston terrier, bullmastiffs, and English and French bulldogs (2–4). A locus on canine chromosome (CFA) 26 has been strongly associated with glioma risk across multiple dog breeds, with regional mapping revealing single nucleotide variants in three neighboring genes *DENR, CAMKK2*, and *P2RX7* that are highly associated with glioma susceptibility (10). The *CAMKK2* and *P2RX7* genes are relevant to the development or progression of human cancers (10). Further characterization of these genetic associations may provide insight into the drivers of gliomas in dogs and humans, identify new therapeutic targets, or decrease the incidence of gliomas in dogs through selective breeding strategies.

## Pathophysiology and Clinical Signs

PBT are intracranial mass lesions that cause clinical signs of brain dysfunction by directly invading or compressing brain tissue and secondarily by causing peritumoral edema, neuroinflammation, obstructive hydrocephalus, and intracranial hemorrhage (11). Compensatory autoregulatory mechanisms, such as decreased cerebrospinal fluid (CSF) production and shifting of CSF into the spinal subarachnoid space, are effective at maintaining the intracranial pressure within physiologic ranges in the early phases of tumor growth. For slow-growing tumor types, such as meningiomas, intracranial pressure-volume homeostatic regulatory mechanisms can often remain intact despite large tumor volumes associated with significant mass effect. However, with progressive increases in tumor volume, autoregulatory mechanisms are eventually overwhelmed and intracranial hypertension (ICH) develops. ICH and the resulting cerebral hypoperfusion is the common pathophysiologic denominator underlying many of the mechanisms of tumor-associated brain injury. Acute clinical deterioration observed in animals with brain tumors and ICH is often the result of vasogenic edema, obstructive hydrocephalus, brain ischemia or hemorrhage, brain herniation, or combinations of these mechanisms (11).

A brain tumor should be considered a differential diagnosis in any middle-aged or older dog with a clinical history consistent with brain dysfunction, especially when clinical signs are progressive. Seizures are the most common clinical manifestation of intracranial neoplasia, and are observed in ~50% of dogs with prosencephalic tumors (3, 12–17). Structural causes of epilepsy, such as brain tumors, should also be suspected in dogs that experience a new onset of seizure activity after 5 years of age, particularly in predisposed breeds (14). Risk factors for tumor-associated structural epilepsy identified on MRI scans in dogs include the presence of tumor involving the frontal lobe, falcine or subtentorial brain herniations, and marked contrast enhancement of the tumor (16). The pathophysiology of tumor-related epilepsy is currently poorly understood, but both the tumoral and peritumoral microenvironments may contribute to epileptogenic phenotypes owing to disordered neuronal connectivity and regulation, impaired glial cell function, and the presence of altered vascular supply and permeability (18–20). Central vestibular dysfunction is the most common clinical sign associated with brain tumors originating in the caudal brainstem (14, 21). Dogs with brain tumors may also present with non-specific clinical signs, such as lethargy, inappetence, and weight loss (22). Tumors in the fronto-olfactory region are often associated with only historical evidence of brain disease such as seizures or behavioral changes and a normal interictal neurological examination.

Most PBT in dogs occur as solitary mass lesions, and tumors involving forebrain structures are more common than those in the brainstem (3, 4, 21). In many cases with solitary masses, the neurological deficits observed reflect the focal neuroanatomic area of the brain containing the tumor. However, dogs with PBT or SBT may also present with neurological deficits indicative of multifocal intracranial disease.

Multifocal clinical signs may result from several phenomena. The tumor or its secondary effects (vasogenic edema, brain herniation, or hemorrhage) may involve more than one region of the brain, which has been reported in up to 50% of dogs with solitary PBT (3). The phenotypes of some PBT, such as butterfly glioblastomas, diffuse glioma, or leptomeningeal oligodendrogliomatosis, are characterized by invasion of both cerebral hemispheres or diffuse brain or meningeal involvement (23–26). Multiple discrete tumor foci may also be present, which occurs occasionally in canine meningiomas and histiocytic sarcomas (1, 27). Rare reports describing synchronous PBT of different histologies, and concurrent PBT and SBT also exist (28, 29). PBT, and particularly choroid plexus carcinomas, may metastasize within the CNS by a unique mechanism termed drop metastases (8, 11, 30). This involves exfoliation of cancer cells into and circulation within the CSF, with implantation of tumor foci distant from the site of the primary tumor in the ventricular system or subarachnoid space.

## Diagnosis of Intracranial Tumors

As brain tumors typically affect middle-aged to older dogs that may have significant concurrent disease, performance of a complete blood count, chemistry profile, and urinalysis is generally indicated in dogs with suspected brain tumors to evaluate the animal's systemic health status (3). These diagnostics also allow for rational formulation of an anesthetic protocol, as anesthesia is required for the performance of ancillary diagnostics that provide the highest diagnostic yield in dogs with structural brain disease, such as brain biopsy, magnetic resonance [MRI] imaging, and CSF analysis. Other diagnostics indicated prior to anesthesia are dictated by the individual dog's history and physical examination findings.

Radiographs of the thorax and abdominal ultrasound (AUS) should be considered in an attempt to identify concurrent unrelated neoplasia or other significant comorbidities. Studies in dogs with intracranial tumors report finding contemporaneous and unrelated neoplasms in 3–23% of dogs with PBT, most of which involve either the thoracic or abdominal cavity (3, 31). Additional studies investigating the clinical utility of screening thoracic radiography and AUS in dogs with intracranial tumors have indicated that abnormalities are frequently identified on these imaging examinations, the results of these procedures rarely negatively affect the decision to perform neurodiagnostic procedures indicated for the patient's neurological presentation, and significantly alter therapeutic recommendations for the brain tumor in <10% of cases (31, 32).

Cross-sectional diagnostic imaging techniques, such as computed tomography (CT) and MRI have revolutionized the clinical diagnosis and management of brain tumors in veterinary medicine, although MRI is the preferred modality for the assessment of animals with intracranial disease (33–53). Data obtained from MR imaging such as mass number (solitary vs. multiple), origin within the neuraxis (meningeal [extra-axial], parenchymal [intra-axial], or intraventricular) and intrinsic signal appearances, collectively provides characteristic patterns that allow for the presumptive diagnosis of most frequently encountered PBT and SBT in veterinary medicine or refinement of the list of differential diagnoses. The diagnostic imaging features of each of the common types of PBT are reviewed with their comparative neuropathologic features below. In one investigation of 40 dogs, the accuracy of predicting the type of PBT based on MR images was 70% (44). It remains common in veterinary practice to make clinical decisions in patients with presumptively diagnosed tumors based on imaging derived data, despite the potential consequences this has on management of individual patients. There are also significant implications associated with the strength of evidence provided by studies that include cohorts of dogs without histopathologic tumor diagnoses.

Cerebrospinal fluid (CSF) analysis generally provides data that is complementary to the clinical examination and diagnostic imaging results and may assist in the prioritization of differential diagnosis. Obtaining CSF in dogs with brain tumors and intracranial hypertension carries a risk of causing clinical deterioration. Although this risk is low, it should be critically assessed in each patient and evaluated in context of the likelihood of obtaining a non-specific test result. Advanced imaging of the brain should always precede CSF collection to best evaluate individual patient risk.

While CSF is a sensitive indicator of intracranial disease and is frequently abnormal in patients with brain tumors, white blood cell (WBC) counts, WBC differentials, and total protein concentrations are highly variable and are often non-specific for neoplasia. There are conflicting data in the literature about the cytological characteristics of CSF in dogs with meningiomas, with one study reporting that meningiomas were commonly associated with a modest neutrophilic pleocytosis, and a later investigation concluding that the majority of meningiomas have WBC counts <5 cells, and a neutrophilic pleocytosis was an atypical finding for canine meningiomas located in the rostral or middle fossas (54, 55). In one study of CPT, CSF analysis was helpful for the differentiation of CPC from CPP, as observation of a CSF total protein concentration >80 mg/dL was exclusively associated with a diagnosis of CPC (8). Exfoliated neoplastic cells may be observed in the CSF cytology of dogs with any type of brain tumor, but CPT, lymphoma, and histiocytic sarcoma are the tumors most commonly reported to be detected with CSF analysis (3, 8, 56–58).

An evolving field in comparative medicine is the identification of circulating biomarkers in blood or CSF that correlate with brain tumor load, biological behavior, or treatment response. Liquid biopsy is a technique for sampling and analyzing non-solid biological tissues, and is mainly used to diagnose disease or monitor therapy. Serum or plasma compounds such as VEGF, glial fibrillary acidic protein, plasma free amino acid profiles, and CSF concentrations of uric acid, D-dimers, and MMP-2 and MMP-9 have been quantified in dogs with brain tumors, but results to date suggest that these compounds will not fulfill ideal biomarker criteria due to limitations associated with sensitivity and specificity (59–65).

Circulating tumor DNA (ctDNA), circulating cell-free RNA, serum proteomic profiling, and exosomes are also currently being explored as liquid biopsy biomarkers in veterinary neurooncology (66, 67). Serum proteomic microarray profiles classified dogs as having intracranial neoplasia, meningoencephalitis of unknown etiology, or being healthy with 100% accuracy and preliminary data suggests that proteomic immunosignatures may also have utility in distinguishing tumor types (67). A feasibility study in canine glioma has demonstrated that expression of cell surface integrins αvβ3 and α3β can be detected in canine glioma cell lines, as well as exosomes derived from these lines using integrin specific peptide targeting methods (66). This investigation provides the foundation for further exploration of serum and CSF exosomes as potential *in vivo* biomarkers of canine glioma.

Given the limitations of imaging, CSF analyses, and liquid biopsy techniques, histopathologic examination of representative tissue is required for the definitive diagnosis and grading of nervous system tumors. Excisional biopsy performed during curative intent surgery remains the most frequently employed biopsy technique for PBT in veterinary practice, but these procedures have been historically limited to patients with superficially located, extra-axial forebrain tumors. In cases where surgical resection may not be a possible or optimal approach to management, stereotactic brain biopsy (SBB) techniques are often a viable alternative method for establishing a histopathologic diagnosis.

Several SBB techniques have been utilized in dogs with brain tumors including endoscopic assisted, free-hand, and image-guided procedures. SBB associated diagnostic yields, morbidity, and mortality are variable and dependent on the technique used, the neurologic status of the patient, experience of the surgeon, and the neuroanatomic location of the lesion (68–74). However, similar to the human experience, when SBB is performed in dogs with brain tumors by an experienced veterinary neurosurgeons, diagnostic yields are 95%, and serious adverse events occur in <5% of cases (72, 74). The histological classification and grading of CNS tumors can be challenging, especially when evaluating the limited sample sizes associated with SBB, but accurate diagnoses can be facilitated by taking multiple biopsy specimens (72, 74).

## Glioma

### Diagnostic Imaging Features

**Figure 1:**
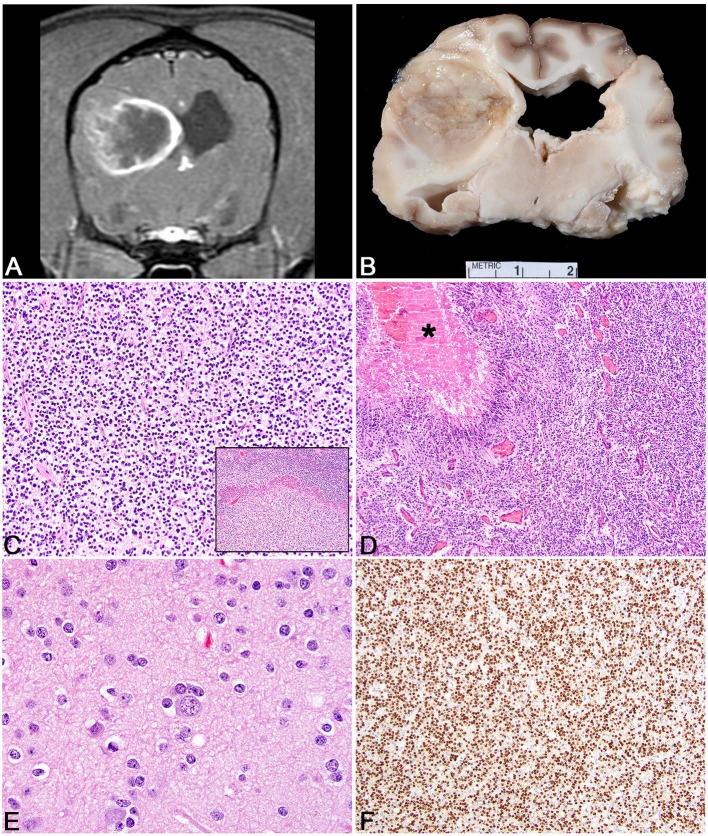
Glioma. **(A)** Canine; MRI-High-Grade Oligodendroglioma. Transverse, post-contrast T1W image illustrating heterogeneously hypointense and ring-enhancing intra-axial mass in the right parieto-temporal lobes. The mass is markedly attenuating the right lateral ventricle. **(B)** Canine, High-Grade Oligodendroglioma. Corresponding necropsy specimen of **(A)** illustrating a well-demarcated mass is present in cerebral cortex causing compression of the thalamus and dilation of the lateral ventricles. **(C)** Canine, High-Grade Oligodendroglioma. Sheets of neoplastic oligodendrocytes embedded in a loose matrix. Hematoxylin and eosin (HE). Inset: Areas of microvascular proliferation. HE. **(D)** Canine, High-Grade Astrocytoma. Discrete areas of necrosis (asterisk) with pseuopalisading by neoplastic cells. HE. **(E)** Human, Grade II Oligodendroglioma. Abundant peri-neuronal satellitosis (secondary structure) is present in this neoplasm. HE. **(F)** Canine, High-Grade Oligodendroglioma. Diffuse intranuclear immunolabeling for Olig2 immunohistochemistry (IHC).

Gliomas originate within the brain parenchyma (intra-axial). As gliomas may infiltrate or displace the neuroparenchyma, they may appear poorly or well marginated and may or may not demonstrate contrast enhancement (Table 1) (33, 44, 46, 51, 75). Among contrast enhancing gliomas, the patterns and degree of enhancement seen can be highly variable. A “ring enhancing” pattern, in which a circular ring of contrast enhancement surrounds non-enhancing abnormal tissue, is often associated with gliomas (Figure 1A). However, ring enhancement is a non-specific finding that has been associated with several neoplastic, vascular, and inflammatory brain diseases (35, 49). Using conventional MRI sequences, it is not currently possible to reliably differentiate types of gliomas (astrocytomas from oligodendrogliomas) or accurately predict the grade of gliomas. However, contrast enhancement is more commonly observed in high-grade compared to low-grade gliomas, and oligodendrogliomas are more likely to distort the ventricles and have contact with the brain surface than astrocytomas (47, 50, 75). The significant overlap that exists in the imaging features of gliomas, cerebrovascular accidents, and inflammatory lesions results in frequent misdiagnosis of these categories of intra-axial lesions (40, 49). The addition of diffusion weighted (DWI), spectroscopic, and perfusion weighted imaging sequences to conventional MRI sequences improves the ability of MRI to discriminate between neoplastic and non-neoplastic lesions, and prediction of tumor grades. However, the utility of this suite of imaging techniques has not yet been evaluated in large populations of dogs with histologically confirmed lesions (34, 41–43, 45, 48, 53, 76).

**Table 1:** Magnetic resonance imaging features of common canine primary brain tumors.

	**Meningioma** **(3, 7, 36–40, 44, 46, 51)**	**Glioma (35, 37, 39, 40, 43, 44, 46, 47, 49–51, 75)**	**Choroid plexus tumor** **(3, 8, 44, 46, 51)**
**Imaging feature**			
Mass origin	Extra-axial	Intra-axial	Intraventricular; lateral aperture of 4^th^ ventricle
Margination • Well-defined/regular • Poorly-defined/irregular	85% 15%	55% 45%	85% 15%
T1W signal • Isointense • Hypointense • Hyperintense • Heterogeneous	70% 10% 20%	80% 50%	35% 20% 45%
T2W signal • Isointense • Hypointense • Hyperintense • Heterogeneous	24% 1% 75%	90% 60%	10% 90%
Contrast enhancement pattern • None • Partial or complete ring • Moderate/severe	10% 55%-homogeneous 35%-heterogeneous	25% (more likely with LGG) 45% (more likely with HGG) 35%	<5% 95+%
Peritumoral edema • Present • Absent	90% 10%	60% 40%	55% 45%
Mass effect • Present • Absent	90% 10%	85% 15%	65% 35%
Other features	Dural tail (35%) Cysts/ITF (20%)	Ventricular distortion (Oligo) No Cysts/ITF (more likely with LGG)	Ventriculomegaly (75%) Drop metastases (33% CPC)

*CPC, Choroid plexus carcinoma; HGG, high-grade glioma; ITF, intratumoral fluid; LGG, low-grade glioma; Oligo, Oligodendroglioma*.

### Pathologic and Comparative Features

The pathology underpinnings of canine glioma have undergone much development in the last several decades with advances in histologic interpretation, immunohistochemical profiling, and molecular diagnostics. Of the total glioma number, oligodendrogliomas have an incidence close to 70% with astrocytomas ~20%, and undefined 10%. Grossly, oligodendrogliomas vary from well-demarcated white to tan, fleshy, soft masses (Figure 1B) to those that are more infiltrative in the neuroparenchyma. They are commonly gelatinous owing to a high level of mucin production. They can be found intraventricularly, multifocally within the central nervous system, or diffusely in the leptomeninges as either a primary mass or metastatic spread (77–79). Astrocytomas tend to be similar in color to the adjacent neuroparenchyma and often blend with the adjacent parenchyma without obvious mass formation (78). Undefined gliomas have a population of astrocytic and oligodendroglial differentiation that are roughly equivalent and therefore the gross features can include any or all of those noted above. For all of the three glioma subtypes in the dog, higher grade tumors are more likely to have associated hemorrhage. In some instances, calvarial bone erosion can be associated with glioma; however, this is not specific to glioma (80).

Historically, canine gliomas were defined based on the original World Health Organization criteria; however, this outdated classification scheme has been replaced by a more comprehensive grading system (78, 81). Based on the new histologic grading scheme, gliomas are divided into oligodendroglioma, astrocytoma, and undefined glioma based on their histomorphology (Figure 1C). Neoplasms are further divided into low- or high-grade. High-grade tumors are diagnosed based on any one of the following being present in the tumor sections examined: microvascular proliferation (Figure 1C, inset), intratumoral, geographic necrosis, pseudopalisading around the regions of necrosis (Figure 1D), increased mitotic activity, or overt features of malignancy (78). The degree of infiltration is not a criterion for differentiating low- vs. high-grade; however, the pattern (non-infiltrative, focally infiltrative, or diffusely infiltrative) should be noted when making the diagnosis (78). Readers are urged to review the updated histologic characterization criteria as an in depth discussion of histologic features are outside the scope of this review paper (78). However, generally speaking, oligodendrogliomas are composed of sheets and linear cords of round to polygonal cells with variable amounts of cytoplasm, round nuclei, and a finely stippled chromatin pattern (82). Especially in necropsy or poorly fixed samples, neoplastic cells often have abundant clear cytoplasm leading to a fried-egg appearance. This is a feature absent in biopsy samples due to timely and appropriate fixation. The formation of secondary structures (neuronal satellitosis, leptomeningeal spread, and perivascular proliferation) are commonly noted in canine oligodendroglioma (Figure 1E). Low-grade tumors often have a delicate meshwork of branching vasculature whereas high-grade tumors have marked vascular proliferation with a glomeruloid appearance (78). Other features that can be observed in canine oligodendroglioma nuclear molding, pseudorosettes, mineralization, and microcysts, which all occur regardless of grade (78).

Astrocytomas have distinct histologic features compared to oligodendrogliomas. High-grade astrocytomas are more frequently invasive than low-grade astrocytomas (78). Neoplastic astrocytes generally have variable amounts of eosinophilic cytoplasm, elongate to ovoid nuclei, with an open chromatin pattern. Various phenotypic features that can be observed histologically in canine astrocytomas include gemistocytic and pilocytic patterns (83). Similar to oligodendrogliomas, microcysts and mineralization can be observed and while rare deposits of mucin can be noted, they are never as abundant as they are in oligodendrogliomas (78, 82). A seemingly unique astrocytoma phenotypic variant has been observed in the cerebellum/caudal fossa in which tumors are well-demarcated and formed by sheets of gemistocyte-like cells (78). High-grade features are the same as those defined for oligodendroglioma and while the highest grade astrocytoma has traditionally been referred to as a glioblastoma, that term was replaced with the more encompassing high-grade astrocytoma in the most recent classification (78, 84). Undefined gliomas have any of the histologic features described above for oligodendroglioma and astrocytoma; however, the proportions of each are relatively equal thereby preventing classification into one group or the other.

Immunohistochemistry has been widely studied in canine glioma. For basic diagnostic purposes, immunolabeling for the glial cell transcription factor, Olig2, stains canine glioma effectively (Figure 1F). Its pattern of immunolabeling is most robust in oligodendrogliomas as opposed to astrocytic tumors where the staining intensity and percent of positive cells is decreased (78). Immunolabeling for glial fibrillary acidic protein (GFAP) is considered supportive of the diagnosis of astrocytoma whereas immunolabeling for CNPase is considered supportive of the diagnosis of oligodendroglioma (78). While Ki67 has been utilized in canine glioma, its widely variable expression patterns in the face of expected staining patterns (i.e., in a tumor with markedly increased mitotic activity) questions its utility in these tumors. Importantly, all of the markers noted above are known to have marked variation in labeling patterns in necropsy samples (owing to variations in fixation time, autolysis, amount of formalin used, and other factors) and use of these markers in necropsy samples should be interpreted with caution (78). Immunohistochemical studies in canine oligodendrogliomas support that these tumors have a dedifferentiated phenotype, one that may arise from an oligodendrocyte precursor cell, perhaps specific to the subventricular zone (85). Some of the markers used to support the origin from oligodendrocyte precursor cells include SOX10, nestin, NG2, and doublecortin (85). In addition, canine gliomas express several neuronal markers including synaptophysin supporting an origin from a multipotential cell type (82). Utilization of tissue microarrays of canine glioma for immunohistochemistry has revealed overexpression of EGFR, PDGFRα, IGFBP2 with EGFR expression moderately correlated to Ki67 immunoreactivity (86, 87). Neither COX-2 nor c-kit have been shown to be expressed in canine glioma (88). While isocitrate dehydrogenase 1 (IDH1) expression has been confirmed in canine glioma via immunohistochemistry, further genome analysis has failed to reveal consistent mutations within the gene as opposed to human gliomas where IDH1 mutation is a common finding (89). Lastly, the inflammatory microenvironment has begun to be explored in canine oligodendroglioma with neoplasms having a robust population of infiltrating T lymphocytes and macrophages (typically with a dendritic cell morphology) with far fewer B lymphocytes (90). Increased numbers of regulatory T cells and dendritic cells have also been recorded in the peripheral blood of canine glioma patients (91). Canine gliomas express PD-L1 which may directly relate to the inflammatory milieu present in these patients (91).

Our understanding of the molecular pathogenesis of canine glioma has also greatly expanded in the last few years. Cell lines generated from canine glioma support multipotentiality in these tumors (92). A wide-ranging genomic profiling of canine glioma has been occurring in parallel with the histologic reclassification of canine glioma. This molecular analysis has confirmed some previous reports in the literature and revealed frequently occurring mutations in the p53 pathway, CDK4, CDKN2A, EGFR, and PDGFRA (93–95). PDGFRA is an especially attractive driver mutation as it is expressed in neural stem cells and known to be critical to gliomagenesis; however, it remains to be determined how it exactly exerts its effects in the development of glioma. Specific to the p53 pathway, TP53 protein is commonly recognized in canine astrocytic tumors compared to oligodendrogliomas with more variable expression of MDM2 and p21 noted across the other glioma tumor types (93). Phosphorylated members of the PI3K/AKT/MTOR and RAS/MAPK-pathways are seen more commonly in astrocytic tumors than oligodendrogliomas or undefined gliomas (93, 96). When canine and human glioma molecular signatures are compared, it is clear that there is an abundance of heterogeneity amongst these two species as it relates to gliomagenesis (97). 1p/19q co-deletion, which is common in human gliomas, is absent in the dog (94, 95). Similarly *Tert* promotor mutations are lacking and while R132 mutations of IDH1 have been noted rarely in canine glioma, they do not appear to have a cancer-promoting effect like they do in human glioma (94, 95). Much work remains in determining the molecular pathogenesis of canine glioma and its application to glioma prognosis in the dog.

Histological classification of human gliomas has had a long history (98). Yet each iteration that was based solely on microscopic morphology suffered from intra- and interobserver variability that contributed to prognostic imprecision and negatively impacted treatment decision-making (99, 100). Indeed, microscopic discrimination of astrocytomas from oligodendrogliomas has long proven difficult, even for experienced neuropathologists (101, 102).

The 2007 World Health Organization (WHO) classification was the last to rely solely on microscopy to subdivide tumors into astrocytomas, oligodendrogliomas, or (mixed) oligoastrocytomas. Grading was based on morphological features such as mitoses, microvascular proliferation, and necrosis. Their presence correlated strongly with aggressive biology and permitted diagnostic refinement into seven distinct prognostic entities (98). Comprehensive molecular analyses have transformed contemporary tumor classification schemes. Studies have clearly established that significant intertumoral molecular heterogeneity exists among each of the morphologically-defined diffuse gliomas (103, 104).

Discovery of mutations in isocitrate dehydrogenase 1 (*IDH1*), which encodes a metabolic enzyme involved in the tricarboxylic acid cycle, significantly altered the trajectory of diagnostic neuropathology and laid the groundwork for the 2016 WHO classification update, particularly for lower-grade (WHO grades II and III) gliomas (105). Subsequent studies identified mutations in either *IDH1* or *IDH2* in over 70% of lower-grade gliomas, as well as a small subset (~5%) of secondary glioblastomas that evolved from lower-grade precursors (106). IDH1/2 mutations were found in both astrocytomas and oligodendrogliomas and portended a better outcome that IDH1/2-wild-type (wt) tumors.

Oligodendrogliomas have been known for several decades to feature a recurrent genetic mutation, loss of chromosomes 1p and 19q, which correlates with improved prognosis and response to therapy (107, 108). Comprehensive genomics and integrative bioinformatics studies solidified its role in contemporary human glioma classification. A population-based study that analyzed 3 molecular markers (1p/19q, IDH1/2, and TERT promoter) stratified grade II and III gliomas into five molecular subgroups independently associated with clinical outcomes (109). A TCGA lower-grade glioma study used comprehensive multiple omics data and unbiased integrative bioinformatics analyses to define three molecular subtypes based on two molecular markers, 1p/19q codeletion and IDH1/2 mutations (110). Importantly, each subtype—IDHmt lacking 1p/19q codeletion, IDHmt with1p/19q codeletion, and IDHwt—were prognostically significant and non-overlapping. Half of patients with 1p/19q-codeleted tumors survived 8.0 years, IDHmt, non-codeleted tumors 6.3 years and IDHwt tumors 1.7 years. Most IDH-mt tumors without codeletion were histological astrocytomas, and nearly all featured mutations in TP53 and ATRX. Most IDH-mt tumors with codeletion were histological oligodendrogliomas and harbored CIC, FUBP1, NOTCH1, and TERT promoter (TERTp) mutations. These data confirmed previous reports identifying CIC and FUBP1 as candidate oligodendroglioma tumor suppressor genes lost on chromosomes 1p and 19q respectively (111). Other large studies have reported similar findings (112, 113).

A significant fraction of WHO grade II/III gliomas lack IDH mutations, especially grade III tumors with astrocytic histology (114). These tumors frequently harbor molecular alterations typically seen in WHO grade IV glioblastomas, including chromosome 7 gains, chromosome 10 deletions, *EGFR* amplification, TERTp mutations, and deletions of *CDKN2A*. The prognosis of these IDHwt tumors is worse than their corresponding IDHmt counterparts. The unfavorable prognosis is particularly strong for IDHwt anaplastic gliomas (114). In this study, IDHwt tumors were further divided into a molecularly unfavorable group (those having either EGFR amplification, H3F3A mutation, or TERTp mutation) and a favorable group lacking those alterations. A subsequent TCGA analysis of both lower-grade glioma and glioblastoma datasets showed that IDHwt lower-grade gliomas segregated into three DNA methylation subtypes (115). Two harbored glioblastoma-like mutations, including EGFR, PTEN, and NF1. The third methylation subtype shared mutational similarities to the non-diffuse glioma, pilocytic astrocytoma (WHO grade I), and portended a similarly favorable prognosis.

Based largely on these studies, the 2016 WHO classification of human gliomas represented a nosological shift in focus away from diagnoses based solely on morphological criteria to one of integrative diagnoses based on both phenotype and genotype (116). Six diagnostic entities were established, each with a required molecular marker (Table 2). With increased interest in canine glioma and larger molecular studies being performed in these tumors, it is logical that molecular classification will be layered onto the newly revised histologic classification to create a more detailed stratification of canine glial tumors.

**Table 2:** Current classification scheme of human glioma.

**WHO grade**	**2007 WHO**	**2016 WHO**
II	Diffuse astrocytoma Oligoastrocytoma Oligodendroglioma	Diffuse astrocytoma, IDH-mutant Oligodendroglioma, IDH-mutant and 1p/19q codeleted
III	Anaplastic astrocytoma Anaplastic oligoastrocytoma Anaplastic oligodendrogliomas	Anaplastic astrocytoma, IDH-mutant Anaplastic oligodendroglioma, IDH-mutant and 1p/19q codeleted
IV	Glioblastoma	Glioblastoma, IDH-wildtype Glioblastoma, IDH-mutant

## Meningioma

### Diagnostic Imaging Features

**Figure 2:**
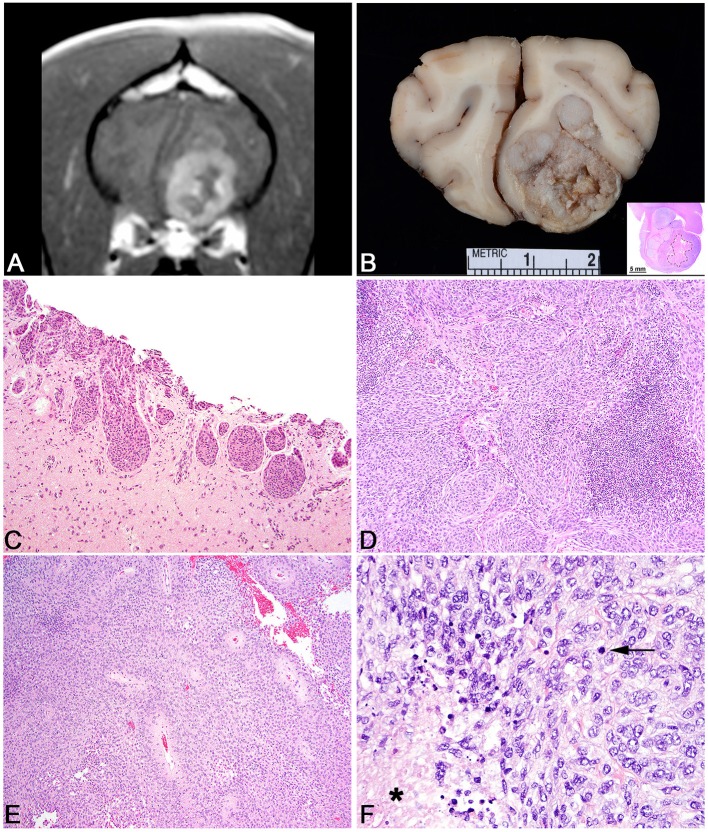
Meningioma. **(A)** Canine; MRI. Transverse, post-contrast T1W image of a well-demarcated, ring-enhancing extra-axial mass in the ventral aspect of the left frontal lobe. Mass effect is present as manifested by the falx shift to the right. **(B)** Canine, Meningioma. Corresponding necropsy specimen of **(A)** illustrating a large, multilobular, white to tan mass that invades the neuroparenchyma. Inset: subgross histologic representation of the meningioma characterized by peripheral sheets of meningothelial cells centered on a core of necrosis. Hematoxylin and eosin (HE). **(C)** Canine, Transitional Meningioma, Invasive. Markedly invasive whorls and clusters of neoplastic meningothelial cells. HE. **(D)** Canine, Transitional Meningioma. Tightly packed whorls and clusters with an associated focus of neutrophils. HE. **(E)** Canine, Meningothelial Meningioma. Sheets of meningothelial cells that surround blood vessels with an acellular perivascular halo. HE. **(F)** Human, Anaplastic Meningioma. Large area of necrosis (asterisk) with sheets of meningothelial cells with increased mitotic figures (arrows). HE.

Meningiomas are the most common extra-axial meningeal origin tumors in dogs (Table 1). Although most meningiomas occur as solitary masses, multiple tumors can be seen (27). Meningiomas usually have a broad-based attachment where they interface with the skull, have distinct tumor margins, and demonstrate marked and often uniform contrast enhancement (Figure 2A). Some meningiomas will also contain intratumoral fluid, large cystic regions, intratumoral mineralization, calvarial hyperostosis, or demonstrate a dural tail sign (36, 38, 46, 51). Although the dural tail feature is commonly associated with meningiomas, it is not specific for this tumor type, or for neoplastic diseases in general (38, 40). Peritumoral edema is present in >90% of canine meningiomas, and is extensive and diffuse in many cases (7, 44, 46). In dogs, reported sensitivities of MRI to correctly identify intracranial meningiomas range between 60 and 100% (3, 35, 37, 39, 44). Histiocytic sarcomas and granular cell tumors share many overlapping imaging features significant with those of meningiomas (46, 51). Specific subtypes of meningiomas cannot be distinguished using conventional MRI sequences (7), although quantitative radiomic analyses has shown some promise in discriminating grades of meningiomas (52).

### Pathologic and Comparative Features

As noted, meningiomas are the most common primary brain and spinal cord neoplasm in the dog. These tumors arise from the arachnoid cap cells that line the middle layer of meninges. They are an intradural, but extraparenchymal solitary neoplasm of which their incidence decreases caudally through the central nervous system, perhaps due to a decreased density of arachnoid cells. Grossly, they tend to be firm, multilobulated, gray to white masses that are compressive and variably infiltrative (Figure 2B). Less common manifestations include a broad, *en plaque* presentation, in which the meningioma spreads over a large area of the brain and is commonly infiltrative into the adjacent neuroparenchyma. This less common pattern most often presents around the ventral portion of the brainstem, especially along the skull base (117). While they can often be locally infiltrative, spread within the central nervous system is not well recorded in the literature and metastatic spread to extra-CNS organs is an exceedingly rare event (118).

Based on their location, they are one of the few primary brain tumors that can be amenable to surgical excision and owing to their complex origin from neural crest cells and mesoderm, they exhibit a vast array of histologic patterns that correlate well to similar patterns seen in human meningioma. The World Health Organization (WHO) histologic classification scheme of canine meningioma recognizes two broad categories: slow-growing, generally benign neoplasms (which represents a number of subtypes: meningothelial, fibrous, transitional, psammomatous, angiomatous, myxoid, granular, and papillary) and more anaplastic tumors which generally don't have as much benign behavior (81). However, it must be stressed that the canine classification scheme is greatly outdated, and much work remains to be done to develop a canine classification scheme that can be correlated with and predict biologic behavior. In comparison, the human classification scheme is vastly more advanced (116). Indeed, many veterinary pathologists have resorted to using the human criteria for grading canine meningiomas due to a lack of adequate canine grading criteria (7). While the 2016 WHO classification of human meningiomas continues to rely solely on microscopic morphology, molecular analyses coupled with detailed histologic analysis are poised to revolutionize classification of these neoplasms. Such advances promise better determination of tumor behavior and patient outcome.

The most common histologic subtypes of canine meningioma are meningothelial, fibroblastic, transitional, and atypical. Meningothelial meningiomas are formed by sheets of meningothelial cells often with vague whorling (Figures 2C,D). Cells typically have abundant cytoplasm, large round to ovoid nuclei with an open chromatin pattern and a single, often prominent nucleolus. The nuclear features are especially well conserved across different subtypes and can be helpful in guiding the diagnosis on less common subtypes. Fibroblastic meningiomas consist of streams of spindle cells with abundant collagen deposition. The transitional subtype combines the feature of the meningothelial and fibrous subtypes such that both patterns are a significant portion of the neoplasm (Figure 2E). A further subtype of the transitional meningioma is the psammomatous meningioma, so named based on the abundant mineralized concretions that are present in the center of meningothelial whorls. This subtype should only be diagnosed if the psammoma bodies are the predominant histologic pattern as small numbers of psammoma bodies can be found in many subtypes of canine meningioma. Meningothelial and transitional subtypes represent anywhere from 20 to 30% or higher of meningioma incidence (119). Microcystic meningiomas are also a relatively common subtype in the dog and have large, variably sized vacuoles that develop in the neoplastic cells that can often coalesce to form large cysts within the tumor (120). In this rare instance, the gross texture of a microcystic meningioma is more fluctuant than the more common firm texture. Importantly, the microcystic subtype should only be designated if this is the predominant pattern as focal to multifocal microcystic change is common in many subtypes of canine meningioma. The granular cell subtype of meningioma consists of large meningothelial cells with abundant periodic acid-Schiff positive granules within the cytoplasm. It remains to be determined if the granular cell variant of canine meningioma is distinct from the intracranial granular cell tumor in the dog; however, it is likely that they represent the same entity as meningothelial whorls can be found in granular cell tumors of the canine CNS with methodical histologic analysis (121).

Other histologic subtypes include rhabdoid meningioma, which has meningothelial cells with abundant eosinophilic cytoplasm and small, peripheralized nuclei. Papillary subtypes are defined by an abundance of pseudorosettes (122). While this can be the predominant histologic pattern, papillary change can also be a focal change within a meningioma. Although suggested by the WHO canine criteria as being a more benign variant, data supports that these are often aggressive subtypes of canine meningioma with a high degree of recurrence (122). This suggests a more malignant behavior for these tumors in the dog, which would be analogous to the human papillary subtype (122). Angiomatous meningiomas are uncommon and represent a meningioma that is highly vascularized. Atypical meningiomas have any of the features noted above, but also have increased mitotic activity (>4 mitoses per ten 400x fields) or have three or more of the following: increased cellularity, increased anisocytosis and anisokaryosis, patterned necrosis, or patternless growth patterns (7). It must be noted, that in canine meningioma, regions of necrosis are common and should not be over interpreted as always indicating an atypical subtype. While the cut off for mitotic count at 4 in ten 400x fields is based on the human WHO meningioma criteria, it remains to be determined if this is an appropriate cut off for canine meningioma or if a better determination of mitotic activity (i.e., Ki67 or AgNOR) is more predictive. Based on some canine studies, the incidence of atypical meningiomas reaches ~40%, a percentage that far outpaces the incidence of human atypical meningiomas (7). Lastly anaplastic meningiomas represent a very uncommon diagnosis in the dog and have greatly exaggerated features of malignancy compared to the atypical variant (Figure 2F).

Other histologic features of importance in the dog include rare deposition of amyloid and exceedingly rare formations of amianthoid fibers (123). Canine meningiomas are widely infiltrated by inflammatory cells including an abundance of neutrophils, either associated with or unassociated with areas of necrosis, large numbers of macrophages and an abundance of lymphocyte subtypes (124). Initial immunohistochemical analysis for interleukin 6 and 8 expression failed to yield significant expression levels; however, RNA transcriptome analysis of canine meningioma revealed overexpression in a number of immunomodulatory genes (125, 126). Chondroid and osseous metaplasia are sporadically seen in various subtypes, although they are most commonly seen in the optic nerve-associated meningiomas.

Owing to their diverse embryologic origin, it is not surprising that canine meningiomas have recorded immunoreactivity to a wide number of antibodies, none of which are specific to the diagnosis of a meningioma. Diagnosis of a meningioma still relies predominately on histologic analysis even though it can be augmented by specific immunohistochemical assays. Canine meningiomas are almost always positive for vimentin and also commonly express laminin, NSE, E-cadherin, CD34, GLUT-1, claudin-1, PGP9.5, pancytokeratin, and S100 (127, 128). The combination of immunolabeling for vimentin, CD34, and E-cadherin has been proposed as being supportive of the diagnosis of meningioma (127). Additional studies on cell adhesion molecules in canine meningioma has revealed that N-cadherin expression is directly correlated with an invasive phenotype (129). This N-cadherin expression is joined by expression of doublecortin at the invading line of the meningioma as well as increased nuclear β-catenin, all three of which may hold prognostic value for more aggressive meningiomas (129). Progesterone receptor expression has been noted in canine meningiomas, where it is inversely related to Ki67 and a better response to radiation therapy (130, 131). Estrogen receptors are less commonly expressed in canine meningiomas (131).

Vascular endothelial growth factor (VEGF) expression has been studied to some detail in the dog. Various isoforms have been identified in the dog and expression of VEGF in both the meningiomas and plasma from affected patients is related to increased tumor malignancy and a poorer prognosis (60, 132, 133). VEGF expression has not been correlated to proliferation indices nor has it been correlated to peritumoral edema (133, 134). Matrix metalloproteinases have also been a common avenue of exploration in the dog. MMP-2 and - 9 are widely expressed in canine meningioma; however, expression is not correlated with peritumoral edema (135, 136). Additionally, tissue inhibitors of metalloproteinases are also expressed in canine meningioma and is highest in papillary subtypes where it and a similar increase in MMP-2 expression may be associated with the more aggressive phenotype seen in this subtype (137). Human-telomerase reverse transcriptase expression has been described in a number of canine meningioma subtypes; however, has not been evaluated in a prognostic setting (138). Somatostatin receptor-2 expression has recently been described in a variety of canine meningioma subtypes, a finding that is supported by RNAseq data analysis of canine meningioma that revealed increased expression of somatostatin receptors (126, 139). The significance of this finding is unclear; however, similar findings have been noted in a number of human tumors, including meningioma. Folate receptor-α is also overexpressed in canine meningioma, as it is in human meningioma (140).

In depth transcriptome-level analysis of canine meningioma remains in its infancy. Analysis of formalin-fixed, paraffin embedded canine meningioma tissue has proven feasible and yielded significant data to suggest a wide variety of intratumoral abnormalities (126). Underexpressed genes included those associated with tumor suppression and cell adhesion among others (126). Real-time reverse-transcription polymerase chain reaction (RT-PCR) studies do not support significant alterations in *NF2* in the dog compared to human cases (141). Western blot analysis has revealed that dogs have decreased expression of tumor suppressor genes *4.1B* and *TSLC1*; however, additional studies are needed to determine the functional significance of these abnormalities (141). Owing to the relative ease of biopsy sampling for many canine meningiomas, the field is primed to better define the molecular pathogenesis of this common canine tumor.

Like gliomas, histological classification of human meningiomas has had a long history (116). However, microscopic morphology remains the foundation of diagnosis and prognostication of these tumors. Indeed, the 2016 WHO classification included only one addition to its 2007 predecessor: the introduction of brain invasion as an independent diagnostic criterion for atypical (WHO grade II) meningiomas (116). This inclusion has significant implications for its usage in canine meningioma as a significant number of canine meningiomas are invasive even without other atypical features. These tumors continue to be stratified into three histological grades (WHO grade I-III) with increased biological aggressiveness and worse prognosis.

The majority of human meningiomas are benign, slow growing, low grade (WHO grade I) lesions. Nine distinct variants of grade I tumors are recognized histologically: meningothelial, fibrous, transitional, psammomatous, angiomatous, microcystic, secretory, lymphoplasmacyte-rich, and metaplastic meningiomas. All have similar prognosis. WHO grade I meningiomas are distinguished from their benign counterparts on the basis of morphological features or patterns. Atypical meningiomas (WHO grade II) feature one of the nine histological patterns listed above, but also harbor increased mitotic activity (>4 mitoses per 10 high-powered fields), brain invasion, or at least three of the following “soft criteria:” increased cellularity, small cell change, prominent nucleoli, sheeting architecture, or necrotic foci. Two histological variants are also considered WHO grade II: clear cell and chordoid meningiomas. Each of these intermediate grade meningiomas portends an increased risk of recurrence (116). The most malignant meningiomas correspond to WHO grade III. These include anaplastic (malignant) meningiomas, which feature overtly malignant cytology (resembling carcinoma, melanoma, or sarcoma) or histological patterns of their lower-grade counterparts with significantly increased mitotic activity (>20 mitoses per 10 high-powered fields). Two histological variants are also considered WHO grade III: papillary and rhabdoid meningiomas. Taken together, these tumors constitute 1–3% of all meningiomas and can be rapidly fatal. Extent of surgical resection is the most significant prognostic factor.

The molecular pathogenesis of meningioma in humans is better understood than in the dog. There is an association between patients that have neurofibromatosis type 2 and the development of meningioma due to variable mutations in the *NF2* gene and the subsequent production of a non-functional protein known as merlin (142). Although NF2 clearly illustrates that there are familial inheritance tendencies for meningioma, the majority of meningiomas in humans occur as sporadic tumors. Non-NF2 associated meningiomas are prone to a wide variety of mutations, many of which remain to be discovered. Known mutations in genes including *AKT1, TRRAF7, KLF4*, and *CDKN2A* predispose to meningioma, including high grade meningiomas in the human WHO grading scheme (143).

## Choroid Plexus Tumors

### Diagnostic Imaging Features

**Figure 3:**
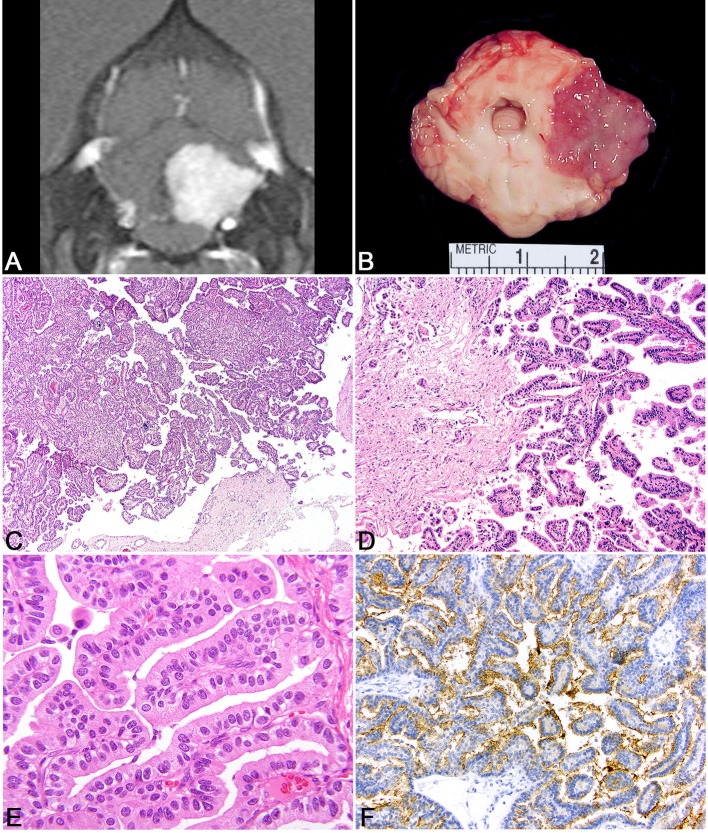
Choroid plexus tumor. **(A)** Canine; MRI. Transverse, post-contrast T1W image demonstrating a uniformly and markedly enhancing, sharply defined mass lesion in the lateral aperture of the 4th ventricle causing compression of the overlying cerebellum. **(B)** Canine, choroid plexus tumor. Corresponding necropsy specimen of **(A)** illustrating a tan, fleshy, slightly granular neoplasm arising at the level of the lateral aperture. **(C)** Canine, choroid plexus tumor. Arborizing trabecular and papillary fronds lined by a single layer of choroid plexus epithelium. Hematoxylin and eosin (HE). **(D)** Canine, Choroid Plexus Carcinoma. Marked invasion into the underlying neuroparenchyma by tubular-like structure of choroid plexus epithelium. HE. **(E)** Human, Choroid Plexus Papilloma. Ribbons, cords, and papillary fronds of choroid plexus epithelium. HE. **(F)** Canine, Choroid Plexus Papilloma. Robust surface immunoreactivity for Kir7.1. Immunohistochemistry (IHC).

Choroid plexus tumors are the most common tumors found in an intraventricular location and these tumors types often demonstrate marked contrast enhancement following gadolinium administration (Figure 3A) (Table 1) (8, 46). Other intraventricular tumors that are occasionally or rarely seen include ependymomas, oligodendrogliomas, primitive neuroectodermal tumors, and central neurocytoma (3, 46, 51). The identification of intraventricular or subarachnoid metastatic tumor implants on MRI studies is a reliable means to clinically discriminate Grade III choroid plexus carcinomas (CPC) from Grade I papillomas (CPP) (8). While MRI is sensitive for the detection of intracranial neoplasms, a normal MRI does not rule out a brain tumor. Lymphomatosis and diffuse glioma (gliomatosis cerebri) are notable for their propensity to be occult on imaging studies of the brain.

### Pathologic and Comparative Features

Choroid plexus tumors are intraventricular neoplasms that occur most commonly in the lateral and 4th ventricles, with a decreased incidence in the rest of the ventricular system. Grossly, the tumors are fleshy with a characteristic finely cobblestoned appearance that is gray to tan on section and variably invades the adjacent parenchyma (Figure 3B). Secondary hydrocephalus is a common accompanying feature due to obstruction of the ventricular system. Tumors are divided into papilloma, atypical papilloma, and carcinoma; however, the determination between the two can be difficult depending on the section examined and neoplasms with a benign histologic characteristics can nevertheless have parenchymal and distant spread. No accepted canine grading scheme is currently used and therefore tumors are commonly graded by the WHO human choroid plexus tumor criteria. The diagnosis of carcinoma is best based on the presence of desmoplasia, microvascular proliferation (which is rare in choroid plexus carcinomas compared to gliomas), and other overt malignant features like high mitotic rate, marked atypia, and patterned regions of necrosis (144). While vascular invasion and extra-neural spread are not a feature of choroid plexus carcinoma, they will seed throughout the ventricular system where they are associated with carcinomatosis in the cerebral spinal fluid and can cause a mass effect at sites distant from the primary location (145).

Histologically, tumors typically form papillary and frond-like arborizing cords and trabeculae supported by a variably dense fibrovascular stroma (Figures 3C–E). Less commonly, areas of more sheet-like growth admixed with pseudorosettes can be found in these tumors (146). Unless invasion into the adjacent parenchyma is present, there are generally few changes in the adjacent tissue and often limited to mild gliosis and edema. Hemorrhage is not typically a feature of this neoplasm. Cells are arranged in simple to multilayered trabeculae with a variable chromatin pattern and typically a single, often inapparent, nucleolus. The mitotic count as determined by histologic examination as well as immunolabeling with Ki67 is highest in carcinomas (144).

While the histologic appearance of these tumors is generally characteristic, they can be mistaken for a metastatic carcinoma on biopsy. Immunohistochemical detection of Kir7.1, an inwardly rectifying potassium channel that has been shown to be a marker for human choroid plexus tumors, will stain the apical portion of neoplastic choroid plexus cells in the dog and can be used as a specific marker to differentiation choroid plexus tumor origin from other carcinomas that may have metastasized to the central nervous system (Figure 3F) (147). Pan-cytokeratin (CKAE1/3) expression is diffuse in choroid plexus tumors (148). E-cadherin and β-catenin labeling are commonly observed in canine choroid plexus tumors and can be associated with aberrant cytoplasmic or nuclear localization (149). While E-cadherin labeling does not vary between benign and malignant CPTs, the related protein N-cadherin has been shown to be localized more commonly to choroid plexus papillomas than carcinomas (150). Immunolabeling for glial fibrillary acidic protein (GFAP) has also been recorded in canine CPTs; however, this is not a specific marker of choroid plexus cells (149). Vascular endothelial growth factor (VEGF), platelet-derived growth factor and it associated receptors, PDGFRα, and PDGFRβ exhibit labeling commonly in CPTs regardless of the designation of papilloma or carcinoma (144). Doublecortin labeling has not been recorded in these tumors (150).

The underlying molecular pathology of canine choroid plexus tumors remains poorly understood and an area ripe for further study. In the largest study to date, comparative cytogenic analysis of human and canine choroid plexus tumors revealed chromosomal losses in a number of chromosomes, including regions that included genes for TP53 and (151). Additional studies including regions that encode for tumor suppression and chromosomal instability genes transcriptome analysis of CPTs to normal canine choroid plexus are needed to better elucidate the underlying molecular mechanisms of these tumors and their potential as a comparative oncology model.

Choroid plexus tumors are uncommon in humans, although they are most commonly observed in children. As noted above, the histologic criteria are defined based on the WHO classification scheme which divides these tumors into three variants: papilloma, atypical papilloma, and carcinoma. Due to the uncommon nature of these tumors, most human studies concentrate on the histologic and immunohistochemical features of these tumors and genomic analysis is less well characterized although a significant number of choroid plexus carcinomas harbor *TP53* mutations (116).

## Contemporary Treatment of PBT in Veterinary Medicine

Studies regarding the treatment of canine brain tumors are frequently limited by the inclusion of presumptively diagnosed cases, variable survival definitions and end-points, lack of inclusion of control groups, no reporting of objective imaging-based criteria of therapeutic response or other quantitative follow-up metrics, retrospective study designs, and small case numbers (152–154). Thus, there currently exists a considerable void in the literature with respect to clinically relevant data regarding tumor type-specific therapeutic outcomes for canine PBT, as well as a lack of adequately powered and rigorously designed trials comparing treatment modalities.

### Palliative Care and Watchful Monitoring

The goal of palliative care is to improve the quality of life of patients and their caregivers through the identification, assessment, and treatment of pain and other physical or behavioral manifestations of the brain tumor. The primary pharmacologic palliative therapies administered to brain tumor patients are anticonvulsant drugs (ACD) for tumor-associated structural epilepsy, corticosteroids targeting peritumoral vasogenic edema, and analgesics for signs consistent with somatic, visceral, or neuropathic cancer pain (21, 152, 153). Although seizures are one of the most common clinical signs associated with brain tumors, ideal ACD protocols for the treatment of tumor-associated epilepsy are currently unknown (3, 21). Phenobarbital, levetiracetam, and zonisamide are popular clinical ACD monotherapy choices. Multidrug ACD protocols are frequently needed to control refractory seizures in dogs with PBT, and the aggressiveness and diligence of the clinician's approach to seizure management is often as important to the therapeutic outcome as the patient's tumor response to other treatment modalities.

Animals that have peritumoral vasogenic edema on MRI are more likely to respond favorably to corticosteroid treatment. However, animals without significant edema may benefit also from the anti-inflammatory and euphoric effects of corticosteroids, and corticosteroid therapy alone may also transiently reduce the tumor volume in some cases (21, 152). Corticosteroids may also provide benefit to animals with tumors causing secondary obstructive hydrocephalus, although surgical CSF diversion via placement of an intraventricular shunt is a more effective method of alleviating clinical signs of intracranial hypertension from obstructive hydrocephalus (155). Polyuria, polydipsia, polyphagia, and sedation are commonly anticipated and reported adverse effects of palliative treatment, but palliative therapies are rarely associated with significant morbidity that necessitates discontinuation of therapy (21, 154).

Pain arising from nervous system tumors can arise from compression or stretching of the meninges, nerve roots, or vasculature, tumor-associated meningitis, neuritis, or radiculitis, and tumor infiltration of the periosteum or musculature. Hyperesthesia of the head or neck is occasionally observed in dogs with brain tumors, being reported in 12% of dogs with PBT in one study (3). Clinical signs consistent with pain often respond to corticosteroid treatment, and additional narcotic or neuropathic pain agents can be added as indicated (156).

Up of 6% canine intracranial meningiomas are identified incidentally (3). Given the increasing clinical usage of serial cross-sectional imaging techniques to manage numerous diseases in veterinary medicine, it is likely that the frequency of identification of incidental brain tumors will increase. Objective observation (e.g., watchful monitoring) represents another reasonable approach to the management of some small and asymptomatic brain tumors. Further characterization of the natural disease history for specific brain tumors will be paramount to identifying optimal indications for watchful monitoring, as well as recommended observation intervals and protocols.

Currently, there is no robust data regarding survival associated with palliative care of specific PBT types or grades. When data for all PBT is pooled, the median survival time (MST) following palliative care is ~9 weeks, with a range of 1–13 weeks (3, 13, 21, 154, 157–159). However, published data does indicate that some dogs with rostrotentorial tumors can experience survivals in excess of 6 months with palliative management (21).

### Chemotherapy

Data evaluating the efficacy of systemically administered chemotherapies for the management of brain tumors in dogs is significantly limited by the lack of definitive tumor diagnoses in the vast majority of reported cases, variable dosing regimens, and a preponderance of small case series examining specific drugs (158, 159). The most commonly used chemotherapeutics for brain tumors are the alkylating agents lomustine (CCNU), carmustine (BCNU) and temozolomide (TMZ), or the antimetabolite hydroxyurea, all of which penetrate the blood-brain-barrier (BBB) (158–160). One retrospective study in 71 dogs with presumptively diagnosed brain tumors reported that lomustine treated dogs (*n* = 56, MST 3 months) experienced no survival benefit compared to dogs receiving palliative therapy (*n* = 15, MST 2 months) (158). In another study conducted in dogs with intra-axial mass lesions (presumptively diagnosed gliomas), dogs treated with lomustine (*n* = 17, MST 4.5 months) survived significantly longer than dogs receiving palliative care only (*n* = 23; MST 1 month) (159). In general, the prognosis is poor for dogs with PBT treated with chemotherapy when used as a monotherapy in the setting of gross disease. No difference in survival was also reported between groups of dogs with presumptively diagnosed gliomas treated with stereotactic radiotherapy with (*n* = 20, MST 13.5 months) or without (*n* = 22, MST 12.4 months) TMZ (160).

At the dose intensities reported in the literature, toxicities associated with CCNU, TMZ and hydroxyurea occur commonly but are rarely life-threatening or require discontinuation of therapy in dogs with brain tumors (158–160).

There is mechanistic justification for the investigation of the utility of existing and new classes of drugs for targeted use in canine brain tumors. Non-steroidal anti-inflammatory drugs (NSAIDs), particularly COX-2 selective inhibitors, have numerous potential, but largely unexplored uses for the management of brain tumors. In addition to their use for analgesia, NSAIDs can have antitumor, anti-edema, and immunomodulatory effects in humans and dogs (161, 162). Overexpression of COX-2 has been demonstrated in canine meningiomas, and this overexpression can promote cancer cell proliferation, angiogenesis, and peritumoral edema, reduce apoptosis of neoplastic cells, and decrease antitumor immunity (161–163). As VEGF/VEGFR1/2 and PDGFRa are also overexpressed in a variety of canine PBT, administration of VEGF-targeted antiangiogenic agents, such as bevacizumab, or the receptor tyrosine kinase inhibitors masitinib or toceranib phosphate have potential for therapeutic use in brain tumors (60, 86, 133, 134, 164).

*In vitro* investigations have demonstrated that responses to chemotherapeutic agents such as bleomycin, carboplatin, CCNU, irinotecan, and TMZ, as well as mechanisms of chemoresistance observed in canine glioma cell lines parallel those seen in human glioma cell lines (93, 165). Thus, it is reasonable to expect that the inclusion of adjuvant chemotherapy in multimodality canine brain tumor treatment protocols could result in modest clinical benefits, as is generally seen in humans. Metronomic chemotherapy with chlorambucil in conjunction with surgical resection is currently being evaluated in an early phase trial in dogs with gliomas (166). The histopathologic and molecular characterization of canine brain tumors and creation of additional of canine patient-derived tumor cell lines will be paramount to efforts necessary to develop and evaluate brain tumor-specific drug protocols, such as high-throughput drug library screening and the identification of biomarkers that predict chemoresponsiveness.

### Surgery

Benefits associated with surgical resection of brain tumors include the rapid reduction or elimination of the tumor burden, the secondary beneficial effect this has on reducing ICP, and allowing for definitive histopathological diagnosis of the tumor. Numerous variables complicate critical comparisons of studies evaluating the efficacy surgical treatment of specific tumor types including the experience of the surgeon, availability, or standardized protocols for usage of a growing number of operating room technologies that may facilitate resection, and a nearly universal lack of inclusion of quantifiable surrogates of surgical success, such as surgeon- or imaging-based extent of resection (EOR) assessments (167, 168). Given that attainment of wide excisions of brain tumors are generally not possible for margin evaluation, objective evaluation of EOR is currently dependent on MRI-based assessments, which are associated with practical limitations including the need for anesthesia, expense and difficulties distinguishing residual tumor from surgically induced reactive changes in the acute postoperative period (167, 169).

Outcomes associated with surgical management of canine meningiomas are highly variable, reflective of the previously identified operator and technical factors, in addition to the propensity for all grades of canine meningiomas to locally invade brain tissue (7). Most surgical studies of canine meningiomas have included predominantly superficially located rostrotentorial tumors (170–174). Surgical treatment of basilar, cerebellopontomedulary angle, foramen magnum, parasellar, and tentorial meningiomas is technically challenging and has not been frequently reported (173). When standard cytoreductive surgical techniques are used, the average MST for canine meningiomas is ~10 months (170–172, 174, 175). Studies describing the use of techniques or technologies that facilitate removal of infiltrative tumor or intraoperative visualization such as cortical resection, usage of an ultrasonic aspirator, or endoscopic assisted resection in canine meningiomas report superior MST (16–70 months) compared to conventional surgical methods (172, 173, 176). Dogs with meningiomas treated surgically also benefit from the addition of adjunctive radiation therapy (RT), as MST associated with multimodal surgery and RT protocols range from 16 and 30 months (130, 171, 177).

There is relatively little data regarding the surgical treatment of other PBT such gliomas and choroid plexus tumors, although a few individual animals with these tumor types experienced prolonged survivals after surgery (8, 152, 157, 178, 179). Surgical treatment of these tumors is infrequently attempted as approaching and removing them is technically demanding due to their intra-axial or intraventricular locations. In addition, as these tumors are poorly delineated and locally invasive, it is inherently more difficult to discriminate the margins of tumor from the neighboring neural tissue. Outcomes in the current literature suggests that surgery should probably not be used as a sole treatment modality for canine gliomas, as MST for rostrotentorial gliomas treated surgically was 2 months (174). MST of ~6 months have been reported in dogs with gliomas treated surgically in combination with metronomic chemotherapy or immunotherapy (166, 180). Resection of these tumors types is likely to become more common and efficacious with the evolution of brain imaging techniques, intraoperative stereotactic and neuronavigational systems, minimally invasive automated tissue resection devices, and “tumor painting” with fluorophores to assist with intraoperative visualization of tumors (152, 153, 181, 182).

The frequency of significant adverse events associated with surgical treatment of PBT varies widely between individual studies, ranging from 6 and 100% (157, 166, 170–176, 178, 179, 183). Removing the outlier study with 100% perioperative mortality results in an average rate of acute perioperative mortality of ~11%, although nearly 50% of dogs experience acute adverse effects associated with intracranial surgery in studies that focus on adverse event reporting (154, 179, 183). Common causes of morbidity and early perioperative mortality include aspiration pneumonia, intracranial hemorrhage or infarction, pneumocephalus, medically refractory provoked seizures, transient or permanent neurological disability, electrolyte and osmotic disturbances, and thermoregulatory dysfunction (154, 183).

### Radiation Therapy (RT)

RT is beneficial for the treatment of PBT when used as a monotherapy or adjunctive modality. Extrapolating meaningful outcome data from the veterinary brain tumor RT literature associated with specific tumor types in confounded by a general lack of histologically diagnosed tumors included in RT studies, grouping of highly heterogeneous intracranial masses, which sometimes include PBT and SBT in data analyses, and considerable variability in the RT types and dose prescriptions used. RT equipment and techniques have advanced from the early use of orthovoltage RT to current predominantly linear accelerator based options, including advanced forms of intensity-modulated RT (IMRT) such as VMAT and tomotherapy, as well as stereotactic RT (SRT) and stereotactic radiosurgery (SRS) (160, 184).

Cohorts of dogs treated with RT as a sole treatment modality for presumptively diagnosed brain tumors indicate an average survival of ~16 months, with reported overall MST ranging from 7 and 24 months when all treated intracranial masses are analyzed (157, 160, 171, 177, 184–191). Some RT studies reported that extra-axial tumors (meningiomas) have a more favorable prognosis than intra-axial (glial) tumors (157, 187, 191). MST associated with RT treatment of extra-axial masses, the majority of which were presumptively diagnosed meningiomas, range from 9 and 30 months, and MST reported for intra-axial masses range from 7.5 and 13 months (33, 157, 160, 171, 177, 184, 186–191). Given the variable outcomes associated with both RT and surgery, there is currently no clearly superior choice between these two modalities when either is used a sole treatment for canine meningiomas (154).

RT also has utility in the adjunctive therapy of canine meningiomas, with combined surgical and RT therapy producing MST of 16–30 months (130, 171, 177). A select number of molecular biomarkers have been evaluated for their prognostic value in dogs with meningiomas treated with surgery and RT. In one study of 17 dogs treated with surgery and adjunctive hypofractionated RT, survival was negatively correlated with VEGF expression (132). The MST was 25 months for dogs with tumors with ≤75% cells demonstrating immunoreactivity to VEGF compared with 15 months for dogs with tumors with >75% of cell staining for VEGF, and dogs with tumors demonstrating more intense VEGF immunoreactivity also had a significantly shorter survival time (132). In a study that included 70 dogs with meningiomas treated with surgery and hypofractionated RT, tumor proliferative indices were assessed via MIB-1/Ki67 IHC, and were not associated with survival (134). There was also no association between VEGF and MIB-1/Ki67 expression (134). Progesterone receptor expression has also been shown to be inversely related to the tumor proliferative index (PF_PCNA_ index), which was predictive of survival in dogs with meningiomas after surgery and postoperative RT. The 2-year progression-free survival was 42% for tumors with a PF_PCNA_ index ≥24 and 91% for tumors with a PF_PCNA_ index <24% (130).

Approximately 10% of brain tumor cases treated with RT will experience treatment-related mortality or adverse effects (154). Frequently reported adverse effects include aspiration pneumonia, pulmonary thromboembolism, acute CNS toxicity which often manifests as a decreased level of consciousness, damage to at risk organs in the treatment field including deafness, cataract formation, and keratitis, and late-onset radiation necrosis (154). Although significant adverse events associated with SRT and SRS have been uncommonly reported in animals to date, the more frequent use of high-dose per fraction prescriptions may eventually influence the incidence of observed toxicity.

## Novel Therapeutic Approaches

The potential of pet dog dogs with naturally occurring cancers to provide answers to fundamental cancer drug and device development questions that meet a critical and shared need among stakeholders in the cancer research and global healthcare communities is being increasingly realized (192, 193). Translational studies in brain tumor-bearing dogs can provide a variety of safety, pharmacokinetic, mechanistic, toxicity and anti-tumor activity data in an immunocompetent host, and thus offer numerous opportunities to guide the therapeutic development process. The current landscape of comparative veterinary neuro-oncologic research encompasses four primary focus areas: (1) novel, macroscopic tumor targeting, imaging, or CNS delivery techniques, (2) therapeutics molecularly targeting tumor-specific markers or aberrant cellular pathways, (3) immunotherapy, and (4) modifications of the dosing or chemistry of existing or repurposing of therapeutics based on new mechanistic discoveries. Several of these therapeutic approaches have been evaluated in early phase clinical trials in dogs with brain tumors, and numerous others are in the pre-clinical developmental stages.

Macroscopic tumor targeting techniques seek to eliminate of the tumor burden by facilitating gross surgical resection or delivery of ablative energy doses by improving visualization of or sensitizing cancerous tissue, or to overcome therapeutic the drug delivery limitations imposed by the BBB in brain tissue within and surrounding the tumor mass that is visible on imaging studies. The use of intraoperative stereotactic equipment and neuronavigational systems with various tissue resection devices and techniques are being actively investigated for the surgical biopsy and treatment of canine brain tumors (152, 154, 181). The safety and feasibility of stereotactic tumor ablation using lasers and pulsed electrical fields have also been demonstrated in canine brain tumors (194–196). BBB disruptive technologies that allow for CNS drug delivery such as transcranial focused ultrasound and irreversible electroporation are also being used to treat canine brain tumors (195, 197). Convection-enhanced delivery (CED), an approach that bypasses the BBB and allows for direct intratumoral delivery of macromolecular drugs, has been used to evaluate the toxicity and preliminary efficacy of a variety of non-selective and targeted chemotherapeutics, gene therapy, and biodegradable nanomaterial drug carriers in several early phase trials in dogs with brain tumors (152, 153, 197–202). In dogs with intracranial gliomas, studies investigating intratumoral CED of non-selective chemotherapeutics, such as irinotecan (198), and molecularly targeted agents, including EGFRvIII- antibody conjugated magnetic iron oxide nanoparticles (201) and modified bacterial cytotoxins conjugated to IL-13RA2 and EphA2 receptor ligands (197), all have been shown to be capable of inducing significant antitumor effects without major adverse effects.

There is an expanding library of targeted agents being developed for and tested in canine brain tumors, and these agents represent a wide variety of mechanistic approaches including protease-conjugated oncolytic viruses, immunomodulatory microRNAs or small interfering RNAs, immune-checkpoint inhibitors, apoptosis promoters, radiosensitizing agents, and nanoparticular cytotoxic drugs. These compounds have shown promising anti-tumor effects *in vitro* or *in vivo* against non-CNS tumors, the ability to penetrate the BBB when administered systemically, or favorable safety and pharmacokinetic profiles in healthy dogs, and are currently in early phase trials in dogs with tumors (197, 203–206). Proof-of principle studies have demonstrated that both EGFR targeted, doxorubicin loaded minicells (204) and PAC-1, a pro-apoptotic, BBB-penetrant small molecule activator of procaspase-3 are capable of achieving clinically relevant intratumoral concentrations when administered systemically to dogs with naturally brain tumors (205). The promising pre-clinical safety, pharmacokinetic, and antitumor results from the canine EGFR targeted, doxorubicin minicells study subsequently informed the design of a Phase I clinical trial applying this approach in human recurrent glioblastoma patients (204).

Numerous immunotherapy strategies whose unifying goal is to augment the patient's T-cell mediated immune response against cancer cells are being explored for use in companion animal brain tumors (152, 153). IT approaches that involve tumor vaccinations with stimulated patient-derived dendritic cells and autologous tumor lysates combined with toll-like receptor ligand adjuvants or immune-checkpoint inhibitors have demonstrated the safety, feasibility and potential efficacy of IT for use in canine glioma and meningioma (175, 207–209). In dogs with meningiomas, vaccination with an autologous tumor cell lysate combined with synthetic toll-like receptor ligands after cytoreductive surgery increased survival (median 645 days) compared with surgically treated historic controls (median 222 days), and the vaccine was demonstrated to induce tumor reactive antibodies (175). Extending findings observed in rodent models which demonstrated that administration of a CD200 peptide inhibitor overcomes immune tolerance induced by tumor vaccination by increasing leukocyte infiltration into the vaccine site, bolstering cytokine and chemokine production, and enhancing tumor cytolytic activity, this approach was recently evaluted in dogs with high-grade gliomas (209). In this study, gliomas were treated with cytoreductive surgery followed by intradermal injection of a CD200-directed peptide prior to delivery of an autologous tumor lysate vaccine. Dogs receiving the canine-specific CD200-directed inhibitor and the autologous tumor lysate had significanlty longer survivals (median 12.7 months) compared to a historical control group of dogs treated with surgery and autologous tumor lysate (median 6.4 months).

There are currently two studies that have evaluated modified dosing regimens of existing chemotherapeutics or repurposing of drugs specifically using canine models of brain cancer. One *in vivo* study demonstrated that metronomic chemotherapy with chlorambucil and lomustine in combination of surgical resection of canine gliomas was well tolerated and capable of achieving potentially therapeutic intratumoral drug concentrations (166). An additional *in vitro* study using canine glioma cell lines reported that benzimidazole anthelmintic treatment increased depolymerization of tubulin and glioma cell cytotoxicity compared to the controls (210), suggesting that both mebendazole and fenbendazole may be reasonable drug candidates for the treatment of canine glioma especially given their established safety profiles.

Advancements in the fields of veterinary and comparative neurooncology will require a change in current daily practice paradigms in which the biopsy of individual animal tumors prior to treatment becomes the new standard of care. This will facilitate the routine and systematic characterization of the histomorphologic and molecular features of canine brain cancers, the continuing global comparative genomic analyses of human and veterinary nervous system neoplasms, and the hosting of comprehensively annotated clinicopathological and neuroradiological data registries. These needs have been further recognized by the neurooncology community, and have begun to be addressed by several projects being conducted by the NCI Comparative Brain Tumor Consortium (193, 211). The NCI Comparative Brain Tumor Consortium was the driving force behind the categorization of canine glioma and has recently embarked on a similar goal of redefining the pathologic features of canine meningioma (78). These coordinated efforts are crucial to determining any prognostic relevance of tumor grading, objectively defining the impacts of treatment modalities on clinical outcomes associated with specific tumors, and the rigorous design of clinical trials, especially considering the expanding repertoire of targeted agents available for cancer diagnostics and treatment.

## Author Contributions

All authors listed have made a substantial, direct and intellectual contribution to the work, and approved it for publication.

### Conflict of Interest

The authors declare that the research was conducted in the absence of any commercial or financial relationships that could be construed as a potential conflict of interest. The reviewer MRC declared a shared affiliation, with no collaboration, with one of the authors, CRM, to the handling editor at time of review.
